# Neoadjuvant Therapy with Drug Arglabin for Breast Cancer with Expression of H-Ras Oncoproteins

**DOI:** 10.31557/APJCP.2020.21.11.3441

**Published:** 2020-11

**Authors:** Sergazy Adekenov, Ainur Zhumakayeva, Vladimir Perminov, Bakhytzhan Bekmanov, Kayrolla Rakhimov

**Affiliations:** 1 *JSC “International Research and Production Holding “Phytochemistry”, Karaganda, Republic of Kazakhstan. *; 2 *Karaganda Medicinal University, Kazakhstan. *; 3 *Center of the Multidisciplinary Hospital No 3 of the Health Administration of the Karaganda Region, Kazakhstan. *; 4 *Institute of General Genetics and Cytology, Kazakhstan. *; 5 *Asfendiyarov Kazakh National Medical University, Kazakhstan.*

**Keywords:** Arglabin, neoadjuvant therapy, farnesyl transferase

## Abstract

**Backgrounds:**

In breast cancer, blocking of Ras signaling and inhibition of H-Ras is quite promising. H-Ras may become a target for farnesyl transferase inhibitors, and in combination with other immunohistochemical factors it will contribute to the progression of a breast tumor.

**Purpose:**

The aim of this study was to evaluate the effectiveness of neoadjuvant therapy for breast cancer with the inclusion of farnesyl transferase inhibitor, arglabin interfering with the expression and concentration of H-Ras oncoproteins.

**Methods:**

Depending on the presence of H-Ras oncoproteins after Western-blot hybridization, the patients were divided a negative and positive expression of H-Ras groups.

**Results:**

Correlation analysis of methods used for determining the expression ability and concentration of H-Ras oncoproteins (immunohistochemistry and Western-blot analysis) demonstrated substantial statistical relationship Rs=0.71, p=0.03. The H-Ras oncoproteins were absent in patients receiving either “Arglabin” or standard AC regimen. However, in the AC + Arglabin group, there was a varying degrees of positive concentration of H-Ras oncoproteins (Kruskal-Wallis=6.92; p=0.03).

**Conclusion:**

These results indicate that Arglabin attenuates H-Ras oncoproteins expression which is a promising therapeutic target for breast cancer.

## Introduction

Breast cancer (BC) is the most common malignant tumor in women in many countries of the world (Ferlay et al., 2018), including Kazakhstan (Igissinov et al., 2019). Like most other types of cancer, it is a heterogeneous disease and several sub-types have been identified. These subtypes are traditionally determined using standard pathological criteria, including cell morphology, an invasive phenotype, and well-established clinical markers such as estrogen alpha-receptor markers, progesterone receptors, and HER2 expression. Depending on the expression of genes, they were phenotypically generalized into different subtypes, each of which with its own clear pathological criteria and treatment options (Perouet al., 2000; Sorlieet al., 2001).

Unfortunately, a significant proportion of patients receiving specific adjuvant therapy develop relapses. This encourages the search for right treatment regimen for a particular patient and hence it is necessary to identify additional biomarkers, allowing individualization of treatment and satisfactory long-term results (Arteaga et al., 2012; Pat. 2015; Zhumakayeva et al., 2018; Cantwell-Dorris et al., 2011).

Gradually, in regard with the advent of new technologies, it appears that the tumor has specific characteristics that can affect the sensitivity to targeted therapy. Identification of these characteristics and their subsequent transfer to a clinical test, which accurately predicts drug sensitivity, will be of great significance in clinical practice and will allow to individualize the approach to each specific patient with breast cancer (Wiesner et al., 2009; Yerushalmi et al., 2010; Stuart-Harris et al., 2008).

Understanding the relative contribution to the processes of oncogenic transformation from the Ras signaling pathways, the activation of these genes, is a prerequisite for developing a rational design of therapeutic strategies for certain types of cancer. Inhibition or stabilization of Ras - effector interactions to prevent downward signaling can provide better opportunities for therapeutic intervention. However, it is necessary to identify suitable molecules that are effective and specific for mutated proteins in order to minimize the effect on normal RAS signaling. Current therapeutic strategies such as tyrosine kinase inhibitors, farnesyltransferase inhibitors, or downstream kinase inhibitors will probably provide promising results in future (Sugita et al., 2018).

The role of RAS genes in malignant tumors has a multifaceted character, since they are involved in the proliferation of various tumor cells. Modern targeted therapy has already demonstrated successful outcome with RAS-associated colorectal cancer, melanoma, pancreatic cancer (Kovalski et al., 2019).However, it is only the beginning, and in the future it will be necessary to unravel the role of this family in other malignant tumors, including breast cancer.

According to data from earlier reports, H-Ras is the most studied RAS gene, but it gained sufficient popularity after works of (Spandidos DA et al., 1984), in which the expression of H-Ras in breast cancer was studied in more detail.

Later, the authors found a relationship between the high expression of the p21 Ras oncogen in breast cancer with an aggressive course of the disease (Kasidet al., 1987; Sommers et al., 1990).

Analysis of the molecular and genetic features of the pathogenesis of breast cancer accompanied by the clinical studies will amplify the effectiveness of therapeutic methods. Active studies are being carried out on proteins involved in ensuring the high viability of malignant cells and determining the prospects for using such proteins as diagnostically significant biomarkers and molecular targets for using targeted drugs.

## Materials and Methods

The study of morphological material postoperative breast cancer tissue for the determination of H-Ras expression was carried at Karaganda Regional Oncology Center (Multidisciplinary Hospital No. 3 of the Health Administration of the Karaganda Region, Republic of Kazakhstan).

The collection of clinical material: medical card, biological material of patients (postoperative histological blocks) was carried out in accordance with the rules adopted by the Ethical Commission of the Medical University of Karaganda.

*Immunohistochemical study*

At the first stage, all patients underwent a retrospective analysis of the morphological material (breast cancer tissue) of breast cancer patients (n=100) with immunohistochemical verification of tissue markers: ER, PR, Her2neu, Ki-67.Immunohistochemical study of biopsy material before treatment with determination of the expression of H-Ras oncoprotein.

In immunohistochemical studies, histological paraffin sections were prepared 5 μm thick, followed by removal of paraffin, dehydration and finally washed with sodium citrate buffer (PBS, sc-294091, Santa Cruz). Immunohistochemical staining of prepared sections was carried out using an Avidin-Biotin antigen detection system ImmunoCruz^®^ ABC Kit (sc-516216) in accordance with the manufacturer’s instructions. The positive reaction, 3,3-diaminobenzidine tetrahydrochloride was visualized using DAB (sc-24982) as a chromogen. 

The mouse monoclonal antibodies anti-IgG1 were used against the H-Ras protein (sc-29, Santa Cruz) of mouse, rat and human origin, characterized by a positive reaction in the cytoplasm of tumor cells. For negative control, primary anti-H-Ras antibodies were substituted with buffer (PBS) or non-immune anti-IgG1.

The intensity and prevalence of H-Ras expression was assessed using the Remmele and Stegner immunoreactivity scale (IRS).

Expression was evaluated as a percentage of positively stained cells in 10 fields of view per 1,000 cells with a magnification of microscope x 200. Staining on the membrane and cytoplasm of tumor cells was considered positive. Negative immunophenotype was considered as the expression of H-Ras with an IRS score (0-2 points), in ≤ 20% of stained tumor cells.

Depending on the expression of H-Ras, the patients were divided into two groups representing negative and positive expression of H-Ras and compared.

*Western-blot hybridization technique*

The Qproteome FFPE Tissue Kit was used to isolate protein from paraffin sections. (Qiagen, Germany).

The Bradford method was used to determine the protein concentration in the samples. It was determined based on the data on its optical density through the equation of the calibration graph.

Protein separation was performed using polyacrylamide gel electrophoresis (10% PAAG). Ammonium persulfate (0.1%) and TEMED (0.1%) were used for gel polymerization. The electrophoresis was carried out in vertically arranged plates (12x12x0.1 cm) for 1-1.5 hours at a voltage of 180V and at a temperature of + 4ºC in the refrigerator. A single Tris-glycine buffer (pH = 5) was used in electrophoresis.

Primary monoclonal antibodies H-Ras (Invitrogen, USA) and secondary Goat anti-Mouse IgG antibodies (Invitrogen, USA) with horseradish peroxidase were used. Beta actin monoclonal antibody (Invitrogen, USA) was used for internal control. 

The H-ras oncoproteins was determined by Western-blotting and electrophoresis was performed in the Laemmli system in a 12% PAAG gel. The separated proteins from the gel were transferred onto a nitrocellulose membrane Immobilon-P (Merck Millipore, USA) on a Bio-Rad Mini Trans-Blot Electrophoretic Transfer Cell apparatus (Bio-Rad, USA) for 16 hours at a current density of 0.2 mA/cm^2^. A buffer [25 mM Tris, 40 mM glycine and 10% ethanol (cathodic) and 300 mM Tris and 10% ethanol (anodic)] was used. It was followed by blockade of membrane for overnight with a solution of skimmed milk powder (5%) in TBST buffer (50 mM Tris-HCl, pH 7.5, 150 mM NaCl, 0.1% Tween-20).Then incubated with primary monoclonal antibodies H-Ras (dilution 1:2,000) for 2 hours at a temperature of + 4°C.After that, the membrane was washed with TBS buffer 3 times for 10 minutes and TBST buffer with milk powder (5%) once for 10 minutes. The membrane was incubated with secondary antibodies to mouse IgG conjugated with horseradish peroxidase (dilution 1:10,000) for 1 hour at room temperature. It was washed 3 times in TBS buffer for 10 minutes and treated for 5 minutes with hydrogen peroxide, coumaric acid and luminol mixture (1,25mMluminol,0,68mMcoumaric acidand0,01%Н_2_О_2_).After that, the membrane was exposed with an X-ray film (Blue Basic Autorad Film 8×10, ISC BioExpress, USA) for 1-3 minutes.

*Neoadjuvant therapy of breast cancer*

Our study included a retrospective analysis of the neoadjuvant treatment of breast cancer patients (n=100) between 2012 and 2014. A blind randomized study was conducted to select a neoadjuvant therapy regimen and assign it to treatment groups.

*Drug therapy was carried out according to one of the following regimen*

The patients (n=38) received AC therapy (Doxorubicin 60 mg/m^2^ and cyclophosphamide 600 mg/m^2^) intravenously once for every 21 day cycle.

Neoadjuvant monotherapy with drug “Arglabin” was carried in patients (n=31) at a dosage of 370 mg/m^2^ from days 1 to 7 of every 21 day cycle.

The AC + Arglabin regimen chemotherapy was performed on patients (n=31) in which doxorubicin 60 mg/m^2^, cyclophosphamide 600 mg/m^2^ given intravenously once and arglabin 370 mg/m^2^ from day 1 to 7 of every 21 day cycle.

Two weeks after the completion of neoadjuvant therapy, the direct effectiveness was assessed by the response criteria of solid tumors RECIST: Complete response (disappearance of all lesions), Partial response (reduction of the sum of lesions diameters by at least 30%), Overall efficiency was assessed as the sum of the total and partial response. Progressive disease-an increase of 20% or more of the sum of the diameters of the main lesions (more than 5 mm), the appearance of one or more new lesions. Stable disease - all other cases.

*Statistical methods*

To describe the quantitative indicators, the mean value and standard deviation in the M ± SD format were used; several independent samples were compared using the non-parametric Kruskal-Wallis test; Spearman’s rank correlation coefficient method (Rs), Fisher’s exact test. Statistical data processing was performed using Statistica 10 application software packages. The level of statistical significance was secured at the level of error probability p<0.05.

## Results

Immunohistochemical determination of the expression of H-Ras oncoproteins has revealed 45% of positive expression and 55% of negative expression of the studied proteins (Table 1).

Using Western-blot hybridization, H-Ras oncoproteins expression was positive in 43% of patients. Among them 13% of patients had strong expression (more than 51% of the concentration of the studied protein in the tumor cell), 13% moderate expression (from 31 to 40% of the protein concentration), 19% had low expression of H-Ras oncoproteins (from 21 to 29% protein concentration). However, in 57% of patients expression of H-Ras oncoproteins was negative (Figure 2).

A comparative analysis of the methods used to determine the H-Ras oncoproteins demonstrated a high association between immunohistochemical study and western blot analysis, despite the differences in the quantitative content of the studied proteins.

Correlation analysis of the methods for determining the expression ability and concentration of H-Ras oncoproteins (immunohistochemistry and Western-blot analysis) a strong statistical relationship Rs=0.71, p=0.03 was obtained (Figure 3).

By Western blot hybridization method, it was found that in the group of patients receiving arglabin therapy and the standard AC regimen (adriablastin + cyclophosphamide), H-Ras oncoproteins were absent, while in the AC + Arglabin group, a mainly positive concentration of H-Ras oncoproteins was observed (Kruskal-Wallis = 6.92; p = 0.03) (Figure 4).

In Western-blot analysis indicators of the immediate effectiveness of neoadjuvant therapy depending on the concentration of H-Ras oncoproteins, AC + Arglabin therapy and monotherapy with Arglabin were most effective (Fisher’s exact test =0.022, p=0.04) (Table 2).

**Figure 1:**
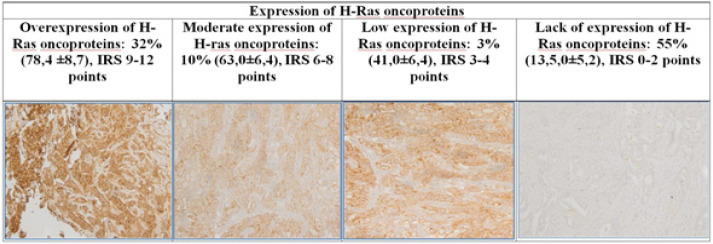
Immunohistochemical determination of H-Ras oncoproteins. 1) Hyperexpression of H-Ras oncoproteins (IRS) 9-12 points in more than 50% of breast cancer tumor cells (staining by the avidin-biotin method); 2) Moderate expression of the H-Ras oncoprotein (IRS) 6-8 points in more than 30% of tumor cells; 3) Low expression of the H-Ras oncoprotein (IRS) 3-4 points in more than 20% of tumor cells; 4) Lack of expression of H-Ras oncoprotein (IRS) 0-2 points - the presence of expression in less than 10% of tumor cells

**Table 1:** Expression of H-Ras Oncoproteins Using Immunohistochemical and Western-Blot Hybridization in Breast Cancer Samples

Methods	H-Ras oncoproteins expression level			
	Strong	Moderate	Low	Negative
Immunohistochemistry	32 (78.4±8.7)	10 (43.0±6.4)	3 (25.3±4.9)	55 (13.5±5.2)
Western-blot hybridization	13 (69.6±8.1)	13 (32.7±2.8)	19 (25.8±2.2)	57 (8.5±5.7)

The values Mean ±SD represent H-Ras expression levels of n=100 samples

**Figure 2:**
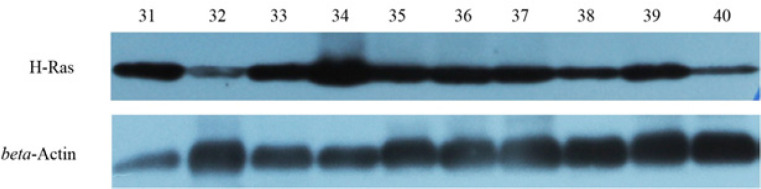
Concentration of H-Ras oncoproteins by Western-blot hybridization. 31-40: Concentration of H-Ras oncoprotein in breast cancer tissue in 10 patients

**Table 2:** Indicators of the Effectiveness of Neoadjuvant Arglabin on H-Ras Oncoproteins

The effectiveness of the treatment	Absence of H-Ras oncoproteins (%)	Positive concentration of H-Ras oncoproteins	р
AC* regimen (n=38)			
Overall effect	79.6 (13.2±4.1)	75.2(51.2±3.2)	0.11
Process stabilization	16.1(12.3±2.1)	18.5 (43.4±4.6)	0.07
Disease progression	4.3 (14.0±1.1)	6.3 (36.0±1.1)	0.12
AC+Arglabin regimen (n=31)			
Overall effect	64.4(14.1±2.4)	86.6%(46.7±3.2)	0.03
Process stabilization	35.6(16.0±0.1)	13.4%(35.4±2.7)	0.02
Disease progression	-	-	-
Arglabin regimen (n=31)			
Overall effect	72.8% (13.2±2.6)	27.2% (69.3±4.1)	0.01
Process stabilization	22.5% (12.0±0.1)	65.5% (52.1±2.1)	0.04
Disease progression	5.5% (11.0±0.1)	7.25% (39.4±2.6)	0.07

AC*, adriablastin + cyclophosphamide; Indicators of the immediate effectiveness of neoadjuvant therapy according to the AC, AC + Arglabin and Arglabin alone represented as % (M±SD)

**Figure 3:**
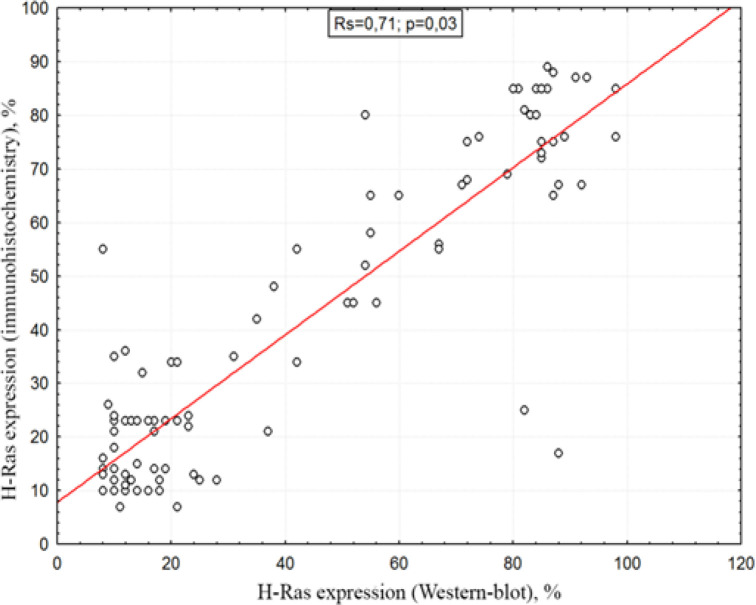
Correlation between Immunohistochemistry and Western-Blot Analysis

**Figure 4:**
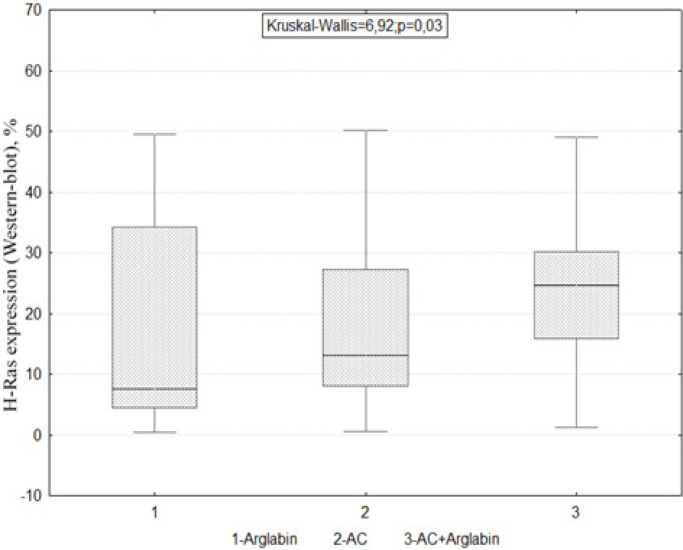
The Effect of Arglabin, AC (Adriablastin + Cyclophosphamide) and Its Combination on the Distribution of H-Ras oncoproteins; Arglabin - 1, AC - 2 and a combination of AC + Arglabin - 3

## Discussion

Most of the studies revealed that the clinical manifestations and molecular profiles of breast cancer are heterogeneous hence significantly affecting the response to the same therapy. A study of H-Ras expression in 297 breast carcinoma patients demonstrated 58% of H-Ras positive tumor cells with high expression of H-Ras oncoproteins (p21ras) which correlated with the largest sized tumor tissue. The presence of regional metastasis and a high proliferative activity index Ki-67, as well as tumors with negative hormonal status were also evident. The detection of positive expression of H-Ras (p21ras) also correlated with a more favorable prognosis in patients without regional metastases in lymph nodes (Sharif et al., 2007).

There are few therapies which have shown encouraging results for example, farnesyl transferase inhibitors appear to be promising H-Ras inhibitors and can facilitate the feedback loop in prior growth factor receptors. Although oncogenic mutations of the RAS, BRAF, and MEK genes are mutually exclusive in this MAPK pathway, combination therapy may be necessary if RAS inhibitors are used in clinics to overcome the concomitant BRAF-MEK mutation (Murugan et al., 2019). Serial biopsies in five patients showed that the activity of the enzyme farnesylprotein transferase was inhibited by ~50% after therapy with tipifarnib (Sparano et al., 2006; Sparano et al., 2009). Likewise, a combination of tipifarnib (200 mg twice every day for 6 days) with an intravenous infusion of doxorubicin+cyclophosphamide (doxorubicin 60 mg/m^2 ^and cyclophosphamide 600 mg/m^2^) for up to four cycles is safe for locally advanced breast cancer. 

In case of luminal breast cancer regimen of letrozole 2.5 mg, 1 tablet 1 time per day + tipifarnib 200 mg 2 times a day, from 1 to 14 days, every 21 days course, showed a high frequency of objective response compared to letrozole monotherapy, with higher overall survival rates (Johnston et al., 2008).

Lonafarnib (SCH66336) inhibits Rheb synthesis and mTOR signaling. In a study of Phase I EORT: lonafanib (SCH66336) combined with trastuzumab and paclitaxel in Her2neu over-expressing breast cancer antitumor activity in 58% of patients and lonafarnib can be safely combined with full doses of paclitaxel and trastuzumab (Taylor et al., 2003). 

At the JSC “International Research and Production Holding “Phytochemistry” (Karaganda, Republic of Kazakhstan), based on dimethylaminoarglabin hydrochloride, the drug “Arglabin” was developed (Patent USA 6,242,617, B1, Jun.5.2001; European Patent № 2069357, 26.08.2015; Eurasian Patent No. 015557 of 30.08.2011; Patent China ZL 2006 8 0055852.4, 26.12.2012 S.M. Adekenov “Method and device for production of lyophilized hydrochloride-1b, 10b-epoxy-13-dimethylaminoguaia-3(4)-en-6,12-olide”).

Our studies showed that Arglabin inhibited farnesylprotein transferase, an enzyme responsible for the formation of malignant tumors (Shaikenov T. E. et al., 2001). The mechanism of action was studied in collaboration with the laboratories of MD Anderson Clinic and Nu Oncology Labs (Houston, USA). It was revealed that Arglabin is a competitive inhibitor of farnesylation of RAS-oncoproteins, reduces the expression of RAS-genes and the content of ATF, causing apoptosis of tumor cells (Shaikenov et al., 1998; Shaikenov et al., 2005; Zhumakayeva et al., 2019).

When studying the effectiveness of therapy in a randomized controlled studies (RCS), breast cancer patients were divided into three groups using the randomization method. The first group patients n = 15 receiving monochemotherapy with Arglabin 6 mg/kg weight or 222 mg/m^2^. The second group consists of patients n = 15, received arglabin in combination with the CMF regimen. The third group patients n =15 received chemotherapy according to the CMF regimen. The highest percentage of partial regression (46.6%) and stabilization (53.3%) was observed in the group receiving a combination of arglabin + CMF, where disease progression was almost absent (Sirota, 2012).

The RCS of Arglabin was also carried out on 92 patients with locally advanced breast cancer of II-III stage, one group of was administered intratumorally with a 2% solution of arglabin at the rate of 2 mg per 1 cm^3^ of the tumor under ultrasound control, the other was administered intravenously with a 2% solution of arglabin based on 5 mg/kg. The effectiveness of standard radiation treatment in patients with locally advanced breast cancer using arglabin was 30% higher than in the control group. When using arglabin against the background of radiation therapy, a uniform tumor regression was observed, and the tumor volume in patients receiving arglabin intratumourly - decreased 5.5 times, intravenously - 8.5 times, and in the control group - 2.8 times (Sirota, 2004).

Currently, there are no clear recommendations on considering the promising information on farnesyltransferase inhibitors in H-Ras expressing breast cancer, there is a dire need for further research and clinical studies before it is marketed.

We evaluated the effectiveness of the neoadjuvant therapy for breast cancer with the inclusion of farnesyltransferase inhibitor Arglabin depending on the expression and concentration of H-Ras oncoproteins.

The determination of molecular and biological markers in breast cancer has two practical meanings: identifying a risk group requiring additional treatment or more careful observation among patients with early stages, or assessing sensitivity to certain types of therapy and individualizing treatment regimens for patients with a common process. The study of new molecular markers can be used in the development of new drugs that target these molecules and block their regulated processes (Zhumakayeva et al., 2019).

When studying the expression of H-Ras by the immunohistochemical method, we determined that 45 (45%) patients had positive staining of the studied proteins. Also, when studying the concentration of H-Ras oncoproteins by Western-blot hybridization, it was found that 43 (43%) patients contain, to varying degrees, a positive concentration of the studied proteins. Given the high correlation between positive expression and the concentration of H-Ras oncoprotein (Rs=0.71, p=0.03), patients were divided depending on the concentration of H-Ras protein in breast cancer tissue into the study group with a positive concentration of oncoproteins (n=43) and a comparison group in which the studied oncoprotein was absent (n=57).

We studied the features of the quantitative distribution of H-Ras oncoproteins depending on the carried out neoadjuvant treatment. It was found that in the group of patients who received Arglabin therapy and the standard AC regimen, H-Ras oncoproteins mostly were not found. In the group of patients with Arglabin monotherapy (n=31), the studied proteins were found in 33.5% (11 patients), while the studied protein was not determined in sufficient concentration in 66.5% (18 patients).Among patients receiving the AC regimen (n=38), the studied protein was absent in 65.8% (25 patients) and 34.2% (13 patients) had a sufficient concentration of H-Ras oncoproteins. In the AC + Arglabin group (n=31), a positive concentration of H-Ras oncoproteins was found in 61.3% (19 patients) and was absent in 38.7% (11 patients) (Kruskal-Wallis=6.92; p=0.03).

Evaluation of the effectiveness of neoadjuvant therapy depending on the concentration of H-Ras oncoproteins showed interesting results. It was found that the most effective treatment regimen was the AC + Arglabin regimen, the frequency of the overall effect with a positive concentration of H-Ras oncoproteins was 86.6% (p=0.03), possibly due to the inhibition of the functional activity of the farnesyltransferase enzyme, and as a result, a decrease activity of H-Ras oncoproteins, which in our opinion led to a more effective regression of tumor tissue and showed statistically significant results. It was also interesting that monotherapy with Arglabin showed a rather high efficiency in the absence of H-Ras oncoproteins, the frequency of the overall effect was 72.8% (р=0.01). There are several arguments in favor of the data obtained, firstly, due to the fact that the farnesyltransferase enzyme is a transport protein and reduces the activity of not only Ras proteins, but also other transport proteins involved in tumor cell proliferation. So at the preclinical stage of studying the mechanism of action of the drug Arglabin, it was shown that it affects the m-ToR signaling pathway, but to a lesser extent than it inhibits the farnesyltransferase enzyme. Perhaps that is why, in the absence of H-Ras oncoproteins, we got quite effective indicators as a result of neoadjuvant monotherapy with Arglabin. The data obtained are new and therefore require in-depth studies in the field of pharmacogenetics.
